# Generalized Entropy Generation Expressions in Gases

**DOI:** 10.3390/e21040330

**Published:** 2019-03-27

**Authors:** Michael H. Peters

**Affiliations:** Department of Chemical and Life Science Engineering, Virginia Commonwealth University, 601 West Main St., Richmond, VA 23284, USA; mpeters@vcu.edu

**Keywords:** entropy generation, entropy generation in gases, entropy flux, statistical mechanics of entropy, second law of thermodynamics

## Abstract

In this study, we generalize our previous methods for obtaining entropy generation in gases without the need to carry through a specific expansion method, such as the Chapman–Enskog method. The generalization, which is based on a scaling analysis, allows for the study of entropy generation in gases for any arbitrary state of the gas and consistently across the conservation equations of mass, momentum, energy, and entropy. Thus, it is shown that it is theoretically possible to alter specific expressions and associated physical outcomes for entropy generation by changing the operating process gas state to regions significantly different than the perturbed, local equilibrium or Chapman–Enskog type state. Such flows could include, for example, hypersonic flows or flows that may be generally called hyper-equilibrium state flows. Our formal scaling analysis also provides partial insight into the nature of entropy generation from an informatics perspective, where we specifically demonstrate the association of entropy generation in gases with uncertainty generated by the approximation error associated with density function expansions.

## 1. Introduction

In general, entropy conservation, including its generation and the concomitant Second Law of Thermodynamics, represents a critical foundation in the analysis of energy efficiency and more broadly in the dictation of both the direction of natural processes and the limitations on its behavior. The specific irreversible nature or “time’s arrow” of nonequilibrium systems are entailed in the so-called phenomenological laws, i.e., Fick’s Law of Diffusion, Newton’s Law of Viscosity, Fourier’s Law of Heat Conduction, and the Law of Entropy Increase. From classical molecular theory, the phenomenological laws of mass, momentum, and energy have been shown to follow from the condition of small perturbations from local equilibrium states [1]. Molecular-based derivations of the conservation equations for mass, momentum, and energy and the associated phenomenological laws have been well-developed based on Irving and Kirkwood’s [2] paper on the “classical” statistical mechanics of transport processes. However, a classical molecular theory derivation of the nonequilibrium entropy conservation equation and the Law of Entropy Increase has remained somewhat elusive or, at best, lacking of comparable clarity and consistency with its sister transport processes. As a disclaimer, no attempt is made here to review the multitude of approaches to the description of nonequilibrium entropy outside the realm of classical statistical mechanics or molecular theoretic approaches, such as coarse graining, quantum approaches, semi-empirical efforts, probability and informatics modeling, phenomenology, etc., which may provide alternative and complimentary paths of analysis. Here we stick to the classical molecular theory approaches that have formed the theoretical foundations of transport phenomena.

Previously [3], we derived the entropy conservation equation in gases following the Irving and Kirkwood approach and using a generalized definition of entropy, which recovers Boltzmann’s entropy in the lowest-ordered gas limit and Gibb’s entropy in the high density limit. Then using a Chapman and Enskog expansion method [4], we obtained an expression for the entropy generation and flux in gases equivalent to that obtained from phenomenology and to Boltzmann’s celebrated expression for entropy generation [3].

In the current study, we refine our approach by obtaining the correct form of entropy generation and flux in gases without resort to the specifics of the Chapman–Enskog expansion method. The formal analysis allows for a general approach or paradigm to the prediction of entropy generation in gases with any arbitrary gas state. This, in turn, allows for the study of entropy generation minimization schemes based on alterations of the gas state beyond the equilibrium perturbation states associated with phenomenology or the Chapman–Enskog expansion type states.

Formal statistical mechanical derivations of the conservation laws begin with the Liouville equation, or the general equation of classical statistical mechanics; so, we must begin there. For the sake of simplicity, we consider only pure component systems here, but the results given are readily extended to multicomponent systems as well.

As noted previously [3], formally the N-particle Liouville equation cannot support finite entropy generation [5,6]. It would be appropriate to refer to this result as the “Liouville entropy paradox”. In the subject of information theory and statistics, entropy is formally defined as a measure of “uncertainty” [7,8,9]. As presented by Jaynes [10], deliberate truncations or approximations to the solution of the Liouville equation are necessary to obtain finite positive entropy generation. As a minimum then, clearly any approximations must be consistent across the transport equations in order to obtain meaningful results. To be clear, the exact origins of the Second Law are not considered here. Rather we seek to develop general expressions to entropy flux and generation in gases based on generalities of perturbation expansions whose approximations lead to finite entropy generation. Our results are shown to be consistent across the transport equations and consistent with phenomenology, and they lead to a general approach for entropy analysis of systems far outside of local equilibrium states. Our formality in scaling and expansions, however, does allow us to partially address the Liouville entropy paradox, as will be shown below.

## 2. Materials and Methods

### 2.1. Liouville Equation and Reduced Forms

In Cartesian vector notation, the Liouville equation for particle number preserving systems reads [11]

(1) $$\frac{\partial f_{N}}{\partial t}=-\sum_{i=1}^{N}\left[\;\frac{p_{i}}{m_{i}}\cdot \frac{\partial f_{N}}{\partial r_{i}}+\;F_{i}\left(r^{N}\right)\cdot \frac{\partial f_{N}}{\partial p_{i}}\right].\;$$

where $f_{N}$ is the N-particle density function, $r_{i}$ and $p_{i}$ are the position and momentum coordinates for the ith particle of mass $m_{i}$, and we have assumed that the force acting on particle *i* is only a function of the position coordinates $r^{N}\equiv (r_{1},r_{2},\ldots ,r_{N}$).

Now consider a reduced form of the Liouville equation for a set of molecules {s}={1,2,3,…,s} that can be obtained by integrating the Liouville equation over the phase-space of the other {N-s} set of molecules. Following standard procedures [11], we can integrate Equation (1) over $dr^{N-s}dp^{N-s}$ space to obtain the *reduced* Liouville equation

(2) $$\frac{\partial f_{s}}{\partial t}+\sum_{i=1}^{s}\;\left(\frac{p_{i}}{m_{i}}\;\cdot \frac{\partial f_{s}}{\partial r_{i}}\right)+\frac{1}{(N-s)!}\;\sum_{i=1}^{s}\frac{\partial }{\partial p_{i}}\cdot \int \int F_{i}f_{N}dr^{N-s}dp^{N-s}=0\;$$

The force acting on the *i*-th molecule can be written as the gradient of a potential as

(3) $$F_{i}=-\frac{\partial }{\partial r_{i}}\Phi^{N}\left(r^{N}\right)\;$$

and for the purposes of specific applications below, the potential will be approximated by a sum over the pair interaction potential as (“pairwise additivity”)

(4) Fi=-∂∂riΦN(rN)≈-∑j=1j≠iN∂∂riϕ(rij)

where the pair potential $\phi (r_{ij})$ is the interaction potential between any two molecules in the system, i.e., the effects of three or more body interactions on the pair potential expression are neglected. Please note that this assumption does not limit the generality of our results given below.

Substituting Equation (4) into Equation (2) and integration on the right-hand side over all the ($r^{N-s},\;p^{N-s}$) space except the (s+1) molecule gives

∂fs∂t+∑i=1spimi·∂fs∂ri-∑j=1j≠is∂ϕ(rij)∂ri·∂fs∂pi

(5) $$=\sum_{i=1}^{s}\int \int \left(\;\frac{\partial \phi (r_{i,s+1})}{\partial r_{i}}\cdot \;\frac{\partial f_{s+1}}{\partial p_{i}}\right)\;dr_{s+1}dp_{s+1}\;$$

Equation (6) is the reduced Liouville equation for pairwise additive interaction forces, and it is an integro-differential equation where the evolution of the $f_{s}$ density depends on the next higher-order, $f_{s+1}$, density. This nonhomogeneous dimensionality feature is the so-called BBGKY hierarchy named after its originators: Bogoliubov, Born, Green, Kirkwood, and Yvon [11].

### 2.2. Entropy Conservation

Irving and Kirkwood derived the mass, momentum, and energy conservation equations from the Liouville equation, and their paradigm will be followed here unless otherwise noted. For more details the reader is referred to the original manuscript [2]. To obtain an entropy conservation equation following the IK (Irving and Kirkwood) approach, we define a general quantity α as [3]

(6) $$\alpha =-k\sum_{s}\left[\frac{N!}{s!\left(N-s\right)!}\right]\frac{1}{s}\sum_{j=1}^{s}\delta \left(r_{j}-r\right)z_{s}\left(r^{s},p^{s},t\right)\;$$

where, following Green [12], $z_{s}$ is the natural logarithm of an *s*-th ordered, normalized density function that depends on the multiparticle expansion method [12,13]. Please note that r is an arbitrary locator vector in the system. The phase-space average of α, <α>, is computed by integrating α over all s phase-space coordinates as

(7) $$<\alpha >=n{\bar{S}}_{BG}=-k\sum_{s}\frac{1}{s!}\frac{1}{s}\sum_{j=1}^{s}\int \int \delta \left(r_{j}-r\right)f_{s}z_{s}dr^{s}dp^{s}\;$$

We call this average the Boltzmann–Gibbs entropy, where ${\bar{S}}_{BG}$ is the entropy per molecule and *n* is the local molecular number density. For s=1, $z_{1}=\ln\left(\hbar^{3}f_{1}\right)$ and we recover Boltzmann’s definition [3,4]

(8) $$n{\bar{S}}_{B}\left(r,t\right)=-k\int f_{1}\left(r,p^{\prime },t\right)\ln\left(\hbar^{3}f_{1}\right)dp^{\prime }\;$$

where *ℏ* is Planck’s constant, and in the limit s=N, it can be shown that we recover Gibbs’ entropy [12]

(9) $$n{\bar{S}}_{G}\left(r,t\right)=-\frac{k}{N!}\frac{1}{N}\sum_{j=1}^{N}\int \int \delta \left(r_{j}-r\right)f_{N}\ln\left(\hbar^{3N}f_{N}\right)dr^{N}dp^{N}\;$$

We note that for systems at global equilibrium $f_{N}$ is independent of the locator vector r and, using the fact that n≡N/V=N/∫dr at global equilibrium, Equation (9) becomes equal to Gibbs’ equilibrium entropy function.

Extensions to other orders can also be shown. Specifically, the nonequilibrium, two-space Green’s entropy would be defined according to Equation (7) as [14]

(10) $$n{\bar{S}}_{2}\left(r,t\right)=-\frac{k}{4}\sum_{j=1}^{2}\int \int \delta \left(r_{j}-r\right)f_{2}z_{2}dr^{2}dp^{2}\;$$

where $z_{2}=\ln\left[f_{2}\left(r^{2},p^{2},t\right)/f_{1}\left(r_{1},p_{1},t\right)f_{1}\left(r_{2},p_{2},t\right)\right]$, which again under global equilibrium conditions can readily be shown to reduce to Green’s two-particle equilibrium expression [14]. Please note that various multiparticle expansion or closure methods for the s-order density functions have been recently studied and reviewed by Singer [13].

Now, the dynamical variable for entropy given above depends explicitly on time through the s-order density functions, whereas the dynamical variables for mass, momentum, and energy introduced by IK have no explicit time dependence. So, to obtain an entropy conservation equation following the IK approach, we can work with the Liouville equation and dynamic variable modified to include the explicit time dependence in α, or it is somewhat easier and equivalent to work directly with the reduced Liouville equation, Equation (6); we choose the latter approach.

Multiplying Equation (6) by

$$-k\sum_{s}\frac{1}{s!}\frac{1}{s}\sum_{j=1}^{s}z_{s}\delta \left(r_{j}-r\right)$$

and integrating over all $\left(r^{s},p^{s}\right)$ space gives the following *entropy conservation equation* [3]

(11) $$n\frac{\partial {\bar{S}}_{BG}}{\partial t}+nv_{0}\cdot \frac{\partial {\bar{S}}_{BG}}{\partial r}=-\frac{\partial }{\partial r}\cdot s+n{\bar{s}}_{g}\;$$

where *n* is the local number density and the bulk or mass average velocity $v_{0}$ is defined by

(12) $$v_{0}\left(r,t\right)\equiv \frac{1}{n}\int \left(\frac{p}{m}\right)f_{1}\left(r,p,t\right)dp\;$$

and the entropy flux vector s also follows as

(13) $$s\left(r,t\right)\equiv -k\sum_{s}\frac{1}{s!s}\sum_{i=1}^{s}\int \int \frac{p_{i}^{{}^{\prime }}}{m_{i}}f_{s}z_{s}dr^{s-1}dp^{s}\;$$

which represents the flux of entropy relative to the bulk average velocity, where $p_{i}^{{}^{\prime }}/m_{i}=p_{i}/m_{i}-v_{0}$.

Now, the last term on the right-hand side represents the entropy generation, specifically

(14) $$n{\bar{s}}_{g}\equiv -k\sum_{s}\frac{1}{s!s}\sum_{i=1}^{s}\sum_{j=1}^{s}\int \int z_{s}\delta \left(r_{j}-r\right)\left[\frac{\partial \Phi \left(r_{i,s+1}\right)}{\partial r_{i}}\cdot \frac{\partial f_{s+1}}{\partial p_{i}}\right]dr^{s}dr_{s+1}dp^{s}dp_{s+1}\;$$

Please note that for s=1, we obtain the well-known Boltzmann’s entropy generation term [11],

(15) $$n{\bar{s}}_{g}\left(r,t\right)=k\int \int \ln\left[\hbar^{3}f_{1}\left(r,p_{1},t\right)\right]\left[\frac{\partial \Phi \left(r_{2},r\right)}{\partial r_{2}}\cdot \frac{\partial f_{2}\left(r,r_{2},p_{1},p_{2},t\right)}{\partial p_{1}}\right]dr_{2}dp_{1}p_{2}\;$$

where we have used Newton’s Third Law in writing this last expression. Please note that further simplification of Equation (14) is possible, but our focus here will be on the s=1 generation term and its particular forms.

### 2.3. Asymptotic Expansions

We now look at the specific expressions for the entropy flux and entropy generation terms based on asymptotic expansions. The truncation of these expansions will be clearly shown to result in finite entropy generation; consequently, the truncations must be consistent across the transport equations for any given system. For the sake of generality, we introduce the following dimensionless variables denoted by an asterisk into the reduced Liouville equation: $t^{*}=t/l_{0}/v_{0}$; $r_{i}^{*}=r_{i}/l_{0}$; $p_{i}^{*}=p_{i}/p_{0}$; $f_{s}^{*}=f_{s}p_{0}^{3s}n_{0}^{-s}$; $F_{i}^{*}=F_{i}/F_{0}=F_{i}\lambda_{0}/\phi_{0}$; where $l_{0},p_{0}\left(\equiv mv_{0}\right)$ and $F_{0}\left(\equiv \phi_{0}/\lambda_{0}\right)$ are characteristic values of length, momentum, and intermolecular interaction force, respectively, where the characteristic force is expressed as a characteristic interaction energy, $\phi_{0}$, divided by a characteristic interaction length scale, $\lambda_{0}$.

Substituting these dimensionless quantities into Equation (5) leads to the non-dimensionless, reduced Liouville equation [14]

∂fs*∂t*+∑i=1spi*·∂fs*∂ri*+1ϵ∑j=1j≠isFi*rij*·∂fs*∂pi*

(16) $$=-\frac{N_{\beta }}{\epsilon }\sum_{i=1}^{s}\int \int F_{i}^{*}\left(r_{i,s+1}\right)\cdot \frac{\partial f_{s+1}^{*}}{\partial p_{i}^{*}}dr_{s+1}^{*}dp_{s+1}^{*}\;$$

where

(17) $$\epsilon =\left(\frac{mv_{0}^{2}}{\phi_{0}}\right)\left(\frac{\lambda_{0}}{l_{0}}\right)\;$$

and

(18) $$N_{\beta }=n_{0}\lambda_{0}^{3}\;$$

are dimensionless groups.

For most problems, the characteristic velocity would be the molecular velocity and, thus, $mv_{0}^{2}∼kT_{0}$, where $T_{0}$ is a characteristic temperature. Also, the characteristic intermolecular potential, $\phi_{0}$, is usually on the order of magnitude of $kT_{0}$, so we can select $\phi_{0}=kT_{0}$ and $mv_{0}^{2}=kT_{0}$ giving

(19) $$\epsilon =\frac{\lambda_{0}}{l_{0}}\;$$

Specifically in Equation (16) above, $l_{0}$ is the length scale over which an appreciable change in $f_{s}$ with respect to $r_{i}$ occurs and $\lambda_{0}$ is the length scale over which an appreciable change in $F_{i}$ with respect to $r_{ij}$ occurs. For example, in most gas and liquid systems $\lambda_{0}<<l_{0}$, and this disparity is used to obtain approximate asymptotic solutions to the reduced Liouville equation. Since appreciable changes in $f_{s}$ are concomitant with changes in its moments (or the transport properties), $l_{0}$ is also referred to as characteristic macroscopic length scale. We will also assume $N_{\beta }$ to be on the order of one for a moderate density gas. The characteristic time is normally associated with the macroscopic scale and $N_{t}$ may be taken to be unity for our purposes here. Please note that the scaling analysis given above is a generalization of that given by Frieman [15].

In both gases and liquids ϵ is a smallness parameter. Under these conditions, the solution to Equation (16) is written in the following regular or Poincare’ perturbation expansion

(20) $$f_{s}^{*}=f_{s}^{*(0)}+\varepsilon f_{s}^{*(1)}+\varepsilon^{2}f_{s}^{*(2)}+...\;$$

and it follows that

(21) $$\ln f_{s}^{*}=\ln f_{s}^{*(0)}+\varepsilon \frac{f_{s}^{*(1)}}{f_{s}^{*(0)}}+O\left(\varepsilon^{2}\right)\;$$

Using the Chapman–Enskog expansion method, it was previously shown that the entropy flux and generation expressions can be obtained from their definitions given above [3]. The expressions include only the so-called kinetic contributions, which is consistent with the s=1 level of analysis. Here we demonstrate a more general method for obtaining the entropy flux and generation expressions. For completeness, we also note that in the case of rarefied and dilute gases the expansion is not “regular”, but can still be readily treated as originally shown by Frieman [14,15]. Also, note that a complete analysis for gases requires both and s=1 and s=2 analysis, the latter of which includes two body interactions and leads to the “potential part” of the flux relationships [11,14]. However, the paradigm given below is readily extended to a complete s=2 analysis and will not be considered here in order to simplify the presentation and results.

For s=1, substitution of Equation (20) into Equation (16) leads to a hierarchy of order equations [14]:

0(1):

(22) $$0=-\int \int F_{1}^{*}\left(r_{12}^{*}\right)\cdot \frac{\partial f_{2}^{*(0)}}{\partial p_{1}^{*}}dr_{12}^{*}dp_{2}^{*}\;$$

0(ϵ):

$$\frac{\partial f_{1}^{*(0)}}{\partial t^{*}}+p_{1}^{*}\cdot \frac{\partial f_{1}^{*(0)}}{\partial r_{1}^{*}}$$

(23) $$-\int \int F_{1}^{*}\left(r_{12}^{*}\right)\cdot \frac{\partial f_{2}^{*(1)}}{\partial p_{1}^{*}}dr_{12}^{*}dp_{2}^{*}\;$$

Now, through straightforward analysis of the s=2 expressions, it can be readily shown that the leading order solution to Equation (22) follows the *local* equilibrium density [14], given in *dimensionless* terms as

(24) $$f_{2}^{*(0)}=\frac{n_{2}^{*(0)}\left(r^{*},r_{12}^{*},t^{*}\right)}{{\left[2\pi T^{*}\left(r^{*},t^{*}\right)\right]}^{3}}exp\left\{-\sum_{j=1}^{2}\frac{p_{j}^{{}^{\prime }*2}}{2T^{*}\left(r^{*},t^{*}\right)}\right\}\;$$

where $p_{j}^{\prime *}$ is the dimensionless momentum of molecule j relative to the bulk motion and $n_{2}^{*(0)}\left(r^{*},r_{12}^{*},t^{*}\right)$ is the dimensionless two particle density function [14]. The relations given above are all that are needed to derive general forms of the entropy flux and entropy generation in gases, as we will now show.

## 3. Results

### Entropy Flux and Entropy Generation

First, we return to *dimensional variables* and expand the logarithm term from Equation (21) for s=1 yielding

(25) $$f_{1}\ln\left(\hbar^{3}f_{1}\right)=f_{1}^{\left(0\right)}\ln\left(\hbar^{3}f_{1}^{\left(0\right)}\right)+f_{1}^{\left(1\right)}\left[1+\ln(\hbar^{3}f_{1}^{\left(0\right)})\right]+O\left(\varepsilon^{2}\right)\;$$

Next, we consider the s=1 entropy flux term $s_{1}$ from Equation (13). Using the expansion Equation (25) and the local equilibrium solution in dimensional terms

(26) $$f_{1}^{\left(0\right)}=\frac{n(r,t)}{{\left[2\pi mkT\right]}^{3/2}}exp\left\{-\frac{p_{1}^{{}^{\prime }2}}{2mkT}\right\}\;$$

Now, using the auxiliary conditions [3]

(27) $$\int f_{1}^{\left(1\right)}dp_{1}=0\;$$

(28) $$\int p_{1}f_{1}^{\left(1\right)}dp_{1}=0\;$$

(29) $$\int p_{1}^{\prime 2}f_{1}^{\left(1\right)}dp_{1}=0\;$$

the entropy flux term can be easily shown to be given by

(30) $$s_{1}\left(r,t\right)=\int p_{1}^{\prime }f_{1}^{\left(1\right)}\left(\frac{p_{1}^{\prime 2}}{2m^{2}T}\right)dp_{1}\;$$

where the leading-order entropy flux term vanishes as required for local equilibrium flows [3]. Noting that the kinetic part of the energy flux relative to the mass average velocity is given to first order in ε by [4,11]

(31) $$q_{k}\equiv \frac{1}{2m^{2}}\int f_{1}^{\left(1\right)}\left(r,p_{1},t\right)p_{1}^{\prime 2}p_{1}^{\prime }dp_{1}\;$$

we see that to first-order in ε we obtain

(32) $$s_{1}=\frac{q_{k}}{T}\;$$

i.e., the first-order entropy flux is equal to the kinetic contribution to the energy flux divided by temperature, as required for this order of approximation.

Now, returning to the entropy generation term, Equation (15), we can substitute the expansions Equations (20) and (21) for s=1, to obtain

$$n{\bar{s}}_{g_{1}}\left(r,t\right)=k\int \int \left[\ln f_{1}^{\left(0\right)}+\frac{f_{1}^{\left(1\right)}}{f_{1}^{\left(0\right)}}+\cdot \right]$$

(33) $$\times \frac{\partial \phi \left(r_{2},r\right)}{\partial r_{2}}\cdot \frac{\partial }{\partial p_{1}}\left[f_{2}^{\left(0\right)}+f_{2}^{\left(1\right)}+\cdot \right]dr_{2}dp_{1}dp_{2}\;$$

Now, we can use the O(ε) equation, Equation (23), in dimensional terms

$$\frac{\partial f_{1}^{\left(0\right)}}{\partial t}+\left[\frac{p_{1}}{m}\cdot \frac{\partial f_{1}^{\left(0\right)}}{\partial r}\right]$$

(34) $$=\int \int \frac{\partial \phi \left(r_{2},r\right)}{\partial r_{2}}\cdot \frac{\partial f_{2}^{\left(1\right)}}{\partial p_{1}}dr_{2}dp_{2}\;$$

for part of the integral term in Equation (33). It can also be readily shown that the leading order terms of Equation (33) all vanish as required for local equilibrium flows [14]. For the derivative terms in Equation (34), we have

(35) $$\frac{\partial f_{1}^{\left(0\right)}}{\partial t}=f_{1}^{\left(0\right)}\frac{\partial \ln n_{1}}{\partial t}+\frac{p_{1}^{\prime }}{kT}f_{1}^{\left(0\right)}\cdot \frac{\partial v_{0}}{\partial t}+f_{1}^{\left(0\right)}\left[\frac{1}{T}\left(\frac{p_{1}^{{}^{\prime }2}}{2mkT}-\frac{3}{2}\right)\right]\frac{\partial T}{\partial t}\;$$

and

(36) $$\frac{p_{1}}{m}\cdot \frac{\partial f_{1}^{\left(0\right)}}{\partial r}=f_{1}^{\left(0\right)}\frac{p_{1}}{m}\cdot \frac{\partial \ln n_{1}}{\partial r}+f_{1}^{\left(0\right)}\frac{p_{1}^{\prime }p_{1}}{mkT}:\frac{\partial v_{0}}{\partial r}+f_{1}^{\left(0\right)}\left[\frac{1}{T}\left(\frac{p_{1}^{{}^{\prime }2}}{2mkT}-\frac{3}{2}\right)\right]\frac{p_{1}}{m}\cdot \frac{\partial T}{\partial r}\;$$

Now, for the $f_{1}^{\left(0\right)}$ gradients, we can use the energy transport equation for equilibrium flows [14] to express the temperature derivatives as

(37) $$\frac{3}{2}kn_{1}\left[\frac{\partial T}{\partial t}+v_{0}\cdot \frac{\partial T}{\partial r}\right]=-p_{k}\frac{\partial }{\partial r}\cdot v_{0}\;$$

where $p_{k}$ is the ideal gas pressure. Finally then, substituting Equation (34) into Equation (33), using Equations (35)–(37) and the auxiliary relations, we obtain *Boltzmann’s level entropy generation* term as

(38) $$n{\bar{s}}_{g_{1}}\left(r,t\right)=-\frac{1}{T}\frac{\partial v_{0}}{\partial r}:\emptyset_{k}-\frac{1}{T^{2}}\frac{\partial T}{\partial r}\cdot q_{k}\;$$

where

(39) $$P_{k}=\int p_{1}^{\prime }p_{1}^{\prime }f_{1}dp_{1}=P_{k}^{\left(0\right)}+\emptyset_{k}^{\left(1\right)}\;$$

is the kinetic contribution to the total pressure tensor, and

(40) $$P_{k}^{\left(0\right)}=\int p_{1}^{\prime }p_{1}^{\prime }f_{1}^{\left(0\right)}dp_{1}=p_{k}I\;$$

is the ideal gas pressure, and the kinetic contribution to the shear stress tensor is defined as [4,11]

(41) $$\emptyset_{k}^{\left(1\right)}=\int p_{1}^{\prime }p_{1}^{\prime }f_{1}^{\left(1\right)}dp_{1}\;$$

Thus, Boltzmann’s entropy generation term recovers phenomenological results [16], where the energy flux q and momentum flux flux tensor P are restricted to their kinetic contributions as required to this order, s=1. For completeness, we note that the nonequilibrium work expression follows from the energy conservation equation originally given by IK.

## 4. Discussion

We have shown that we can readily obtain the specific s=1 order entropy flux and generation terms through a simple ordering analysis without regard to the specifics of the Chapman Enskog method. The results are also shown to be in agreement with phenomenology. The ordering paradigm can be applied to any type of gas flows and therefore opens the possibility of treating systems far removed from local equilibrium states.

It is also enlightening to examine the order of terms appearing in the entropy generation expression obtained. The only surviving term from Equation (33) involves the product of the $f_{1}^{\left(1\right)}/f_{1}^{\left(0\right)}$ term with the $f_{2}^{\left(1\right)}$ term, which are both O(ε) terms. Thus, the only surviving generation term is $O(\varepsilon^{2})$, and it represents the uncertainty generated through the errors in the approximation. Also, it is noted that the resulting physical gradients, as reflected phenomenologically, are of second-order reflecting the higher order character of the generation term. If there were no errors associated with the O(ε) expansion, the generation term would be zero. This latter case would constitute local, reversible gas flows. In the N-particle Liouville analysis, it is assumed that $f_{N}$ is known exactly and hence there is no entropy generation involved. It would be interesting to carry the above analysis for s=1 through to $O(\varepsilon^{2})$ with the errors in the approximation being necessarily of higher order. Indeed, Burnett carried out a partial analysis of the $O(\varepsilon^{2})$ expansion following the Chapman–Enskog method, as reviewed by Chapman and Cowling [4]. Burnett’s results are believed to be applicable to hypersonic flows and several different molecular theory approaches have also been developed [4], but an entropy analysis is lacking.

## 5. Conclusions

A more general paradigm for predicting entropy flux and generation in fluids has been proposed and examined for gases, which opens the door for the treatment of fluids far removed from an equilibrium state. What practical results that can be obtained by attempting to operate fluids in hyper-(far from)equilibrium states remains an unanswered question. Current studies are directed at the s=2 level of analysis as well as a full accounting of $O(\varepsilon^{2})$ expansions, where we seek to understand how approximations play out in higher order.
